# Muscle free amino acid profiles are related to differences in skeletal muscle growth between single and twin ovine fetuses near term

**DOI:** 10.1186/2193-1801-2-483

**Published:** 2013-09-23

**Authors:** Francisco Sales, David Pacheco, Hugh Blair, Paul Kenyon, Sue McCoard

**Affiliations:** Animal Nutrition Team, Animal Nutrition and Health Group, AgResearch, Grasslands Research Centre, Palmerston North, New Zealand; Gravida: National Research Centre for Growth and Development, Auckland, New Zealand; Institute of Veterinary, Animal and Biomedical Sciences, Massey University, Palmerston North, New Zealand; Instituto de Investigaciones Agropecuarias, Centro Regional Kampenaike, Punta Arenas, Chile

**Keywords:** Free amino acid, Fetal muscle, Gestation, Muscle growth, Sheep

## Abstract

Twin sheep fetuses have reduced skeletal muscle weight near birth relative to singles as a result of restricted muscle hypertrophy. Intracellular free amino acids (FAA) are reported to regulate metabolic pathways which control muscle protein accretion, whereby reduced intracellular content of specific FAA may reduce their activation and therefore, muscle hypertrophy. The aim of this study was to determine whether differences in muscle weight between singleton and twin fetuses, under different maternal conditions is associated with reduced concentration of specific FAA. The FAA content in the semitendinosus muscle (ST) in singleton and twin fetuses (rank) at 140 days of gestation from heavy (H) or light (L) ewes fed ad libitum (A) or maintenance (M) level of nutrition was measured. Muscle weight was reduced in twin fetuses compared to singletons in all groups. Reduced concentrations of leucine, threonine and valine, but higher concentrations of methionine, ornithine, lysine and serine were found in twin fetuses compared to singletons. Maternal size and nutrition interaction with rank resulted in reduced glutamine in twins from HM-ewes (H-ewes under M nutrition) compared to their singleton counterparts. Maternal weight interaction with pregnancy rank reduced the concentration of arginine in twins, with a larger effect on H-ewes compared with L-ewes. Maternal size interaction with pregnancy rank resulted in twins from M-ewes to have lower alanine, while twins from A-ewes had lower aspartic acid concentration compared to singletons. The ST muscle weight was positively correlated only with arginine concentration after taking into account rank, size and nutrition. The present results indicate that reduced concentrations of specific intracellular FAA, such as arginine, leucine, valine, glutamine, which are known to play a role in muscle growth, could be acting as limiting factors for muscle hypertrophy in twin fetuses during late gestation. Ewe size and nutrition can influence the concentration of specific FAA in muscle and should be considered in any intervention plan to improve twin fetal muscle growth.

## Background

Increasing prolificacy is an effective way to improve profitability in sheep production systems (Gootwine et al. 2001). However, birth weight, postnatal survival, growth, body composition and lifetime production performance may be reduced as litter size increases (Barker 1998; Greenwood et al. 1998; Morel et al. 2009). Reduced fetal weight near term in twins compared to singletons is associated with decreased skeletal muscle hypertrophy, leading to reduced muscle mass (McCoard et al. 2001). Although maternal undernutrition has a direct effect on fetal and skeletal muscle growth during gestation (Fahey et al. 2005), reduced fetal weight and muscle weight in twins compared to singles is observed even in well-nourished ewes (Freetly and Leymaster 2004). This suggests maternal nutrition is not the only factor to impact fetal and muscle growth as litter size increases.

It is well established that fetal growth is influenced by fetal amino acid (AA) availability (De Boo et al. 2005; Kwon et al. 2004; Liechty et al. 1999). Studies in sheep indicate that the rate of protein accretion in the fetus can be stimulated through the fetal infusion of a mix of AA (De Boo et al. 2005; Liechty et al. 1999). Importantly, AA also have the capacity to signal to metabolic pathways which regulate muscle growth (Brown et al. 2009; Hara et al. 1998) via changes in the intracellular concentration of specific AA (Beugnet et al. 2003; Christie et al. 2002; Sancak et al. 2008). For example, AA signalling plays an important role in the regulation of skeletal muscle hypertrophy in monogastrics, through the activation of specific cell signalling pathways (e.g. mechanistic target of rapamycin, mTOR), which controls protein synthesis (Tan et al. 2009; Yao et al. 2008). However, the potential for specific AA to act as signalling molecules to regulate skeletal muscle hypertrophy during gestation in ruminants is not well understood.

In sheep, we have preliminary evidence (Pacheco et al. 2010) that concentrations of specific intracellular AA in skeletal muscle (e.g. arginine and glutamine), differ between single and twin fetuses in late gestation in nutritionally-restricted ewes. The purpose of this study was to further explore the potential relationship between skeletal muscle mass and intracellular free AA concentration in twins compared to singletons, by testing two hypotheses. Our first hypothesis is that reduced skeletal muscle weight in twin, compared to single fetuses in late gestation, is associated with reduced concentration of specific free AA in muscle, such as glutamine and arginine. Our second hypothesis is, that dam nutrition and body size influence the relationship between skeletal muscle AA and fetal muscle weight, between pregnancy ranks (single vs. twin). To test these hypotheses, the concentration of free AA from the *semitendinosus* (ST) muscle collected from twin and singleton fetuses at 140 days of gestation from heavy and light ewes fed two differing planes of nutrition were compared.

## Results

**Figure 1:**
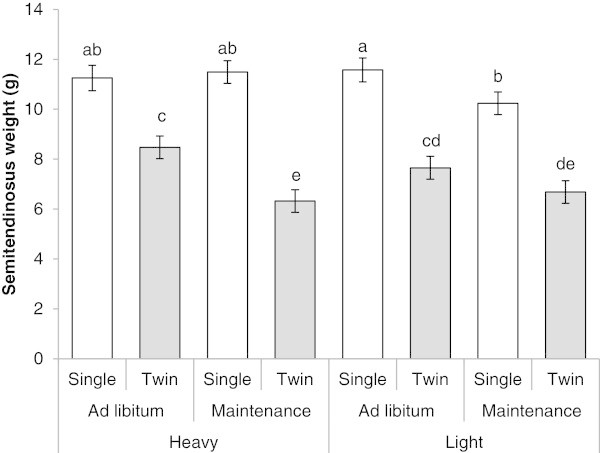
**Fetal** ***semitendinosus*** **muscle weight.** The bars graphic represent the *semitendinosus* muscle weight (g) of the eight groups of fetuses (singletons and twins from heavy and light ewes offered an *ad libitum* or maintenance feeding regime) at 140 days gestation, not adjusted by fetal weight. Bars with different letters are significantly different at *P* ≤ 0.05.

A three-way interaction between pregnancy rank, size and nutrition was observed for ST weight (*P* = 0.04, Figure 1). Twins from HM-ewes had lower ST weights compared with twins from HA-ewes, whereas singletons from LM-ewes had lower ST weight compared with LA singletons (Figure 1). After adjusting for fetal body weight, only a pregnancy rank effect was observed, whereby twins had 17% lower ST weight compared to singles (8.3 ± 0.3 g vs. 10.0 ± 0.3 g, *P* = 0.002). No maternal size, maternal nutrition or interactions with maternal size or nutrition effects were observed for ST weight (data not shown).

A three-way interaction between pregnancy rank, maternal size and plane of nutrition was observed for ST intracellular concentrations of free glutamine and tyrosine (Table 1). Twin fetuses from HM-ewes had 37% lower (*P* = 0.0003) glutamine concentrations compared to their singleton counterpart. Twins from LA-ewes had 32% lower (*P* = 0.02) tyrosine concentration compared to their singleton counterparts. No differences were observed for tyrosine in the other groups.

**Table 1:** **Free amino acid concentration in** ***semitendinosus*** **muscle: three-way interaction**

	Heavy ewes				Light ewes				LSD	***P*** RxSxN
	Ad libitum		Maintenance		Ad libitum		Maintenance			
	Single	Twin	Single	Twin	Single	Twin	Single	Twin		
**Essential amino acids**										
L-Histidine	225	242	201	232	199	261	227	259	76	0.57
L-Leucine	60	52	54	40	50	42	60	47	15	0.96
L-Lysine	109	115	80	81	67	88	70	119	37	0.37
L-Methionine	48	79	42	64	46	86	46	74	17	0.85
L-Phenylalanine	41	50	47	37	44	43	48	47	13	0.18
L-Threonine	1252	823	968	841	1222	839	1212	1065	287	0.82
L-Valine	175	98	119	69	150	121	191	119	38	0.07
**Total EAA**	1,690	1,423	1,455	1,272	1,763	1,462	1,707	1,713	185	0.55
**Non essential amino acids**										
L-Alanine	2393	2495	2239	1858	2137	2164	2400	2164	368	0.55
L-Arginine^1^	410	239	424	263	392	263	340	333	88	0.20
L-Aspartic Acid	465	302	132	85	500	343	423	345	96	0.70
L-Carnosine	1407	1379	1297	1124	1157	1432	1148	1088	323	0.56
L-Citrulline	75	53	59	52	87	72	92	97	27	0.86
L-Cystathionine	140	97	218	157	153	169	287	208	102	0.45
L-Glutamic acid	1483	1240	1191	1201	1045	1807	1365	1835	369	0.14
L-Glutamine	3202^a^	3276^a^	3111^a^	1973^b^	3302^a^	3539^a^	3043^a^	3514^a^	609	0.02
L-Glycine	1844	2794	2010	2868	1888	2215	2134	2052	543	0.56
L-Ornithine	102	99	93	119	69	92	90	113	29	0.32
L-Proline	352	246	370	295	319	285	322	391	105	0.51
L-Serine	1353	1520	995	1474	772	1365	1239	1452	382	0.07
L-Taurine	5501	6046	5786	5770	6170	6728	6485	6939	1348	0.74
L-Tyrosine	51^a^	57^a^	60^a^	52^b^	64^a^	44^a^	68^a^	72^a^	17	0.03
**Total NEAA**	18,634	19,640	17,665	17,238	17,766	19,831	19,361	20,227	895	0.90
**TOTAL**	20,359	21,118	19,177	18,590	19,567	21,358	21,114	22,000	942	0.82

^1^Deemed as conditionally essential (Wu 2009).

Three-way interaction between maternal size,(S, Heavy vs. Light) plane of nutrition (N, Ad libitum vs. Maintenance) and pregnancy rank (R, Single vs. Twin) for the concentration (nmol/g wet tissue) of free amino acids in *M*. *semitendinosus* of fetuses at 140 days gestation. Values are expressed as least square mean (LSM). The average of the difference of the least square means (LSD, α = 0.05) and probability of significance for the three way interaction (RxSxN) are presented.

A two way interaction between pregnancy rank and maternal size was observed for arginine, glutamic acid, glycine and proline in ST muscle (Table 2). Twins from H-ewes had 40% lower (*P* < 0.0001) arginine concentration than their singleton counterparts, whereas twins from L-ewes had only 19% lower (*P* = 0.03) arginine concentration relative to singletons. Twins from L-ewes had 51% higher (*P* < 0.0001) concentration of glutamate compared to singletons, while no difference between pregnancy ranks was observed for fetuses from H-ewes. Twin fetuses had 47% higher (*P* < 0.0001) glycine concentrations compared to singletons in the H-ewes, while no differences were observed between twins and singletons from L-ewes. Twins had 25% lower (*P* = 0.02) proline concentration in H-ewes while no difference between pregnancy ranks was observed for fetuses from L-ewes.

**Table 2:** **Free amino acid concentration in** ***semitendinosus*** **muscle: main effects and two way interactions**

	Rank				Rank x Size						Rank x Nutrition					
	Single	Twin	LSD	***P***	H-S	H-T	L-S	L-T	LSD	***P***	A-S	A-T	M-S	M-T	LSD	***P***
**Essential amino acids**																
L-Histidine	213	249	38	0.07	213	237	213	260	54	0.56	212	251	214	246	54	0.84
L-Leucine	56	45	8	0.01	57	46	55	44	11	0.95	55	47	57	43	11	0.48
L-Lysine	82	101	18	0.04	95	98	69	104	26	0.09	88	102	75	100	26	0.52
L-Methionine	45	76	8	<0.001	45	72	46	80	12	0.39	47	83	44	69	12	0.24
L-Phenylalanine	45	44	7	0.85	44	44	46	45	9	0.94	43	47	47	42	9	0.18
L-Threonine	1163	892	143	<0.001	1110	832	1217	952	203	0.93	1237	831	1090	953	203	0.07
L-Valine	159	102	19	<0.001	147	83	171	120	27	0.49	163	109	155	94	27	0.71
**Total EAA**	1,654	1,467	93	0.05	1,572	1,347	1,735	1,588	131	0.68	1,726	1,442	1,581	1,492	131	0.30
**Non essential amino acids**																
L-Alanine	2292	2170	184	0.19	2316	2176	2268	2164	260	0.85	2265^ab^	2329^a^	2319^a^	2011^b^	260	0.05
L-Arginine^2^	392	274	44	<0.001	417^a^	251^b^	366^a^	298^a^	62	0.03	401	251	382	298	62	0.14
L-Aspartic Acid	380	269	48	<0.001	299	194	461	344	68	0.80	482^a^	323^b^	278^bc^	215^c^	68	0.05
L-Carnosine	1252	1256	162	0.97	1352	1251	1152	1260	229	0.20	1282	1406	1222	1106	229	0.14
L-Citrulline	78	69	14	0.16	67	53	90	84	19	0.52	81	63	76	74	19	0.22
L-Cystathionine	200	158	51	0.11	179	127	220	189	72	0.69	147	133	253	183	72	0.27
L-Glutamic acid	1271	1521	184	0.01	1337^a^	1220^a^	1205^a^	1821^b^	261	<0.001	1264	1523	1278	1518	261	0.92
L-Glutamine^1^	3165	3075	305	0.56	3156	2624	3173	3527	431	<0.001	3252	3408	3077	2743	431	0.11
L-Glycine	1969	2482	272	<0.001	1927	2831	2011	2133	384	0.01	1866	2504	2072	2460	384	0.36
L-Ornithine	89	106	14	0.02	98	109	80	102	20	0.42	86	95	91	116	20	0.30
L-Proline	341	304	53	0.17	361^a^	270^b^	321^ab^	338^ab^	74	0.05	335	265	346	343	74	0.21
L-Serine	1090	1453	191	<0.001	1174	1497	1006	1408	270	0.68	1063	1442	1117	1463	270	0.86
L-Taurine	5985	6371	674	0.26	5644	5908	6327	6834	953	0.72	5836	6387	6135	6354	953	0.62
L-Tyrosine^1^	61	56	8	0.27	55	55	66	58	12	0.33	58	50	64	62	12	0.54
**Total NEAA**	18,357	19,234	448	0.05	18,150	18,439	18,564	20,029	633	0.19	18,200	19,735	18,513	18,733	633	0.15
**TOTAL**	20,054	20,767	471	0.14	19,768	19,854	20,341	21,679	666	0.19	19,963	21,238	20,146	20,295	666	0.24

^1^Refer to Table 1 due to the existence of a three way interaction.

^2^Deemed as conditionally essential (Wu 2009).

Rank effect (Single (S) vs. Twin (T)), two way interaction between pregnancy rank and maternal size (Heavy (H) vs. Light (L)) and between pregnancy rank and plane of nutrition (Ad libitum (A) vs. Maintenance (M)) for the concentration (nmol/g wet tissue) of FAA in *M*. *semitendinosus* of fetuses at 140 days gestation. Values are expressed as least square mean (LSM). The average of the difference of the least square means (LSD, α = 0.05) is presented.

A pregnancy rank by nutrition interaction was observed for alanine and aspartic acid concentration in ST muscle (Table 2). Twin fetuses had 13% lower (*P* = 0.02) alanine concentration compared to singletons in the M-ewes, while no difference was observed between pregnancy ranks in the A-ewes. For aspartic acid, twin fetuses from A-ewes had 33% lower (*P* < 0.0001) concentration compared to singletons from A-ewes, while no difference was observed between ranks in the M-ewes group.

Compared to singletons, twins had lower ST muscle concentration of leucine (19%), threonine (23%), valine (36%) and total EAA (11%) (Table 2), but higher concentrations of methionine (67%), ornithine (19%), lysine (23%), serine (33%) and total non EAA (5%) (Table 2).

**Figure 2:**
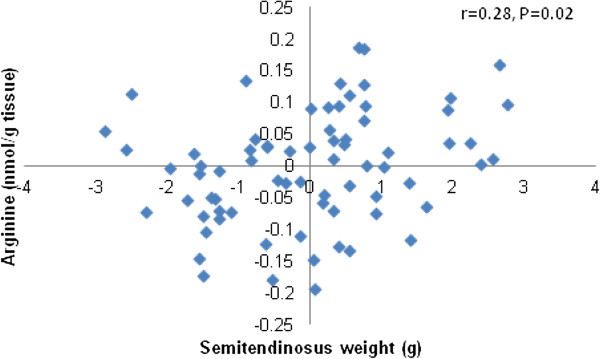
**Partial correlation plot for ST muscle weight with arginine concentration.** The plot graphic shows the partial correlation analysis for ST muscle weight (g) with arginine concentration (nmol/g wet tissue). The analysis considered pooled data of all fetuses and was performed after accounting for the effects of pregnancy rank, maternal size and nutrition.

**Figure 3:**
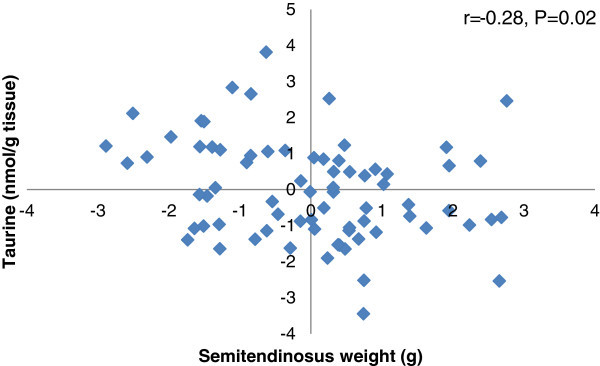
**Partial correlation plot for ST muscle weight with taurine concentration.** The plot graphic shows the partial correlation analysis for ST muscle weight (g) with taurine concentration (nmol/g wet tissue). The analysis considered pooled data of all fetuses and was performed after accounting for the effects of pregnancy rank, maternal size and nutrition.

A positive association was observed between ST muscle weight and arginine concentration after partial correlation analysis (Figure 2). In contrast, a negative correlation was found between ST muscle weight and intracellular concentration of taurine (Figure 3). No correlations were found for any other FAA with ST muscle weight (data not shown).

## Discussion

The objective of this study was to explore the potential relationship between skeletal muscle weight and intracellular FAA concentration in single compared to twin fetuses in late gestation sheep. An additional objective of this study was to establish the effect of maternal size and nutrition on fetal muscle FAA concentration, as an approach to understand some of the possible mechanisms explaining the lower muscle mass normally observed in twin fetuses (McCoard et al. 2001). Reduced ST mass in twins compared to singleton fetuses at 140 days gestation was associated with changes in the concentration of specific but not total intracellular FAA concentration. Notably, the concentrations of arginine, leucine, valine, and glutamine, known to influence pathways which regulate protein synthesis (Wu 2009), were lower in muscle of twin compared to single fetuses. While other AA were affected by pregnancy rank, maternal size or nutrition, their role in fetal muscle growth, beyond being the building blocks for protein synthesis, is unclear. Arginine concentration, irrespectively of pregnancy rank, maternal size and maternal nutrition, was the only AA positively correlated with fetal skeletal muscle mass. These results suggest that arginine may be important for skeletal muscle growth in the late-gestation ovine fetus.

Irrespective of maternal size or maternal nutrition, twin fetuses had lower ST muscle mass compared to singletons, in agreement with a previous study (McCoard et al. 2001). In the present study, the effect of maternal nutrition on muscle mass was influenced by maternal size. Twin fetuses from HM-ewes had disproportionately smaller ST muscle compared to their single counterparts. This indicates that heavy twin-bearing ewes fed a maintenance plane of nutrition were unable to meet the nutritional requirements to maintain not only fetal weight (Blair et al. 2011), but also muscle growth, when compared to their counterparts carrying singletons. Competition for limited nutrients between twins (McCoard et al. 2000) or the reduced priority of nutrient partitioning to fetal skeletal muscle development compared with other organs during maternal nutrient restriction (Du et al. 2010) could explain the lower muscle mass in twins from ewes at a restricted feeding level. In contrast, the lower ST muscle weight observed in singles and twins from LM-ewes, in comparison with their single and twin counterpart from LA-ewes, could indicate that lighter ewes fed a maintenance level of nutrition are unable to provide the nutrient requirements either for a single or twin pregnancy. These results support the notion that skeletal muscle growth in twins is more sensitive to maternal nutritional constraint than in singles, which is in agreement with a previous study (Gootwine et al. 2007). However, our results also suggest muscle growth can be compromised in singleton pregnancies under maternal nutrient restriction, as reported previously (Quigley et al. 2008).

Free AA plays a major role not only as building blocks for protein synthesis, but they also regulate key metabolic pathways which are necessary for cell maintenance and growth (Wu 2009). The function of FAA as signalling molecules is associated with changes in the intracellular concentration of specific FAA (Beugnet et al. 2003; Christie et al. 2002; Sancak et al. 2008). Intracellular pools are therefore critical to accomplish the signalling function of FAA (Nobukuni et al. 2005). The size and composition of the intracellular FAA pool depends on different processes, including availability of circulating FAA, and an increased AA influx or efflux between muscle and the plasma resulting from utilization, (e.g., by protein synthesis) or catabolism (protein turnover) (Hundal and Taylor 2009; Proud 2004). In this study it is unclear what the contribution is of each of these processes to the observed differences in intracellular FAA profiles. Unfortunately, fetal plasma was not available in the present study to relate the plasma FAA profile with intracellular muscle FAA profile in single and twin fetuses. However, we have previously shown that twins from *ad libitum*-fed ewes have lower plasma concentration of glutamine, arginine and leucine compared with singletons at day 140 of pregnancy (van der Linden et al. 2012). Others have also reported a decreased concentration of arginine family members and branched-chain amino acids (BCAA) in fetal plasma when restricting maternal nutrition to 50% of their requirement (Kwon et al. 2004). It has also been proposed that reduced muscle mass in sheep fetuses exposed to maternal nutrient restriction may be associated with reduced plasma FAA, particularly serine, arginine-family AA, and BCAA (Zhu et al. 2006). Therefore, it is feasible that changes in specific FAA concentration in twins muscle were related to changes in circulating AA availability. The possible association between pregnancy rank and maternal nutrition on fetal plasma AA concentration and how this affects intracellular muscle FAA concentrations and muscle mass, is yet to be established.

Skeletal muscle growth in fetuses utilizes both EAA and non-essential AA (NEAA) (Wilkening et al. 1994). However, during fetal stress, such as maternal fasting, AA catabolism increases in muscle, resulting in release to the circulation of gluconeogenic precursors such as glutamine (Liechty and Lemons 1984), due to an increased metabolism of BCAA (Liechty et al. 1987). In the present study, the reduced concentration of the BCAA leucine and valine in ST muscle of twins compared to singletons could suggest higher muscle protein breakdown in twins, resulting in a lower muscle mass compared to singles. In addition, protein breakdown may have also contributed to the observed reduction in glutamine concentration in ST muscle of twin fetuses from HM-ewes. This may have led to the higher growth limitation of muscle from fetuses in the HM group, reinforcing the idea of a greater degree of restriction in this group. Whether the difference in intracellular concentration of FAA is related to changes in FAA transport or a result of catabolism, warrants further investigation.

Arginine is considered a conditionally indispensable AA for the fetus (Wu et al. 2009) and participates in the synthesis of proteins, nitric oxide, polyamines, creatine, some AA and agmatine (Wu and Morris 1998), playing a major role in skeletal muscle growth (Wu et al. 2000). The exacerbated reduction in arginine concentration observed in twins compared to singles from H-ewes compared to their L-ewes counterparts, may be associated with a more stressful fetal environment. Reduced concentration of arginine family members as a result of maternal undernutrition, has been previously described in fetal plasma (Kwon et al. 2004) and in *gastrocnemius* muscle of sheep fetuses (Wu et al. 2006), which supports our findings. The reduced concentration of arginine, could be due to a higher catabolism of this AA, which would explain the increase in ornithine concentration observed in twins compared with singletons, according to Wu and Morris (1998). The higher concentration of arginine found in singletons compared to twins and the positive, although weak partial correlation between intracellular muscle arginine with muscle mass, suggests that arginine may act as a limiting AA for fetal muscle growth in twin fetuses.

Protein synthesis in muscle is controlled by specific signalling pathways, including phosphatidylinositol-3 kinase (Schiaffino and Mammucari 2011), 5′-AMP-activated protein kinase (Bolster et al. 2002) and mitogen-activated protein kinase (Williamson et al. 2003). However, mTOR is accepted as the major pathway regulating muscle protein synthesis (Du et al. 2005). Intracellular FAA can activate mTOR (Beugnet et al. 2003), whereas a decrease in the intracellular AA concentration reduces the mTOR signalling (Sancak et al. 2008). Specific AA such as leucine (Escobar et al. 2006; Suryawan et al. 2008; Suryawan et al. 2011) and arginine (Yao et al. 2008) activate mTOR in muscle of monogastrics, as well as glutamine in cell culture models (Chiu et al. 2012; Nicklin et al. 2009). Preliminary evidence indicates a reduced abundance of mTOR downstream targets in muscle of twin fetuses, compared with singletons at late gestation (Sciascia et al. 2010). Therefore, it is possible that reduced intracellular concentrations of glutamine, leucine and arginine in muscle of twins compared to singles in this study, could have resulted in a decreased mTOR signalling, and therefore, reduced muscle mass. This potential mechanism is part of future research.

The increase in methionine concentration in ST muscle from twins compared to singles is a novel finding. Methionine is an EAA, used to initiate protein synthesis (Kozak 1983); it is involved in DNA methylation (Waterland 2006), participates in oxidative processes (Hoshi and Heinemann 2001) and has other metabolic functions (Brosnan and Brosnan 2006). Previous studies in rats under starvation have shown the increased concentration of methionine in muscle as a result from high protein breakdown (Millward et al. 1974; Millward 1970). However, reduced utilisation from lower protein accretion cannot be excluded.

## Conclusions

Reduced concentrations of specific FAA, which are known to play a role in muscle growth, could act as limiting factors for muscle hypertrophy in twin fetuses in late gestation. The effect of decreased concentration of leucine, valine, glutamine, and especially arginine on fetal skeletal muscle growth in late gestation requires further investigation. The consequences of a maintenance maternal nutrition and maternal weight on fetal muscle mass and concentration of some FAA, reinforces their importance for fetal muscle growth. Altogether, these findings establish a baseline for new studies to further define the role of AA in fetal muscle growth in sheep, and open new possibilities for future strategic nutritional interventions to improve skeletal muscle development.

## Materials and methods

### Animals

All procedures described in the present study were approved by the Animal Ethics Committee of Massey University, Palmerston North, New Zealand.

This study was part of a larger study where animal selection protocols, feeding regimens (Blair et al. 2011; Kenyon et al. 2009; Kenyon et al. 2011) and euthanasia procedures (Blair et al. 2011) were previously described. Briefly, the study design utilised two different maternal sizes, according to their weight and corresponding to the heaviest (H; 60.8 ± 0.18 kg, condition score 3.02 ± 0.03 (1-5 scale (Jefferies 1961)) and lightest (L; 42.5 ± 0.17 kg, condition score 1.97 ± 0.03) Romney ewes, selected from a commercial flock. The ewes were randomly allocated to either a maintenance (M) or *ad libitum* (A) nutritional plane on pasture from day 21 after insemination until 140 days of gestation. Maintenance nutrition was designed to ensure that total ewe live weight increased in pregnancy at a level similar to that of the expected conceptus mass (Rattray 1986; Rattray et al. 1974). The maternal weight change in the maintenance group was coincident with the conceptus mass, which suggest ewes were close to maintenance (Blair et al. 2011; Kenyon et al. 2011). The *ad libitum* plane was designed to provide unrestricted herbage intake under grazing conditions. To achieve these nutritional regimens, ewes were grazed using a rotational system, as described by (Kenyon et al. 2009). Ewes were pregnancy scanned via ultrasound and their pregnancy rank determined (single and twin).

At 140 days of gestation, animals were euthanized and the ST muscle was excised from each fetus, weighed, and snap frozen in liquid nitrogen and stored at −80°C. The numbers of fetuses in each group, according to maternal size, maternal nutrition and pregnancy rank were: HM/single: 10; HM/twin: 10; HA/single: 9; HA/twin: 10; LM/single: 10; LM/twin: 10; LA/single: 10 and LA/twin: 10. The effect of ewe size, plane of nutrition and pregnancy rank on fetal weights have previously been reported by Firth et al. (2008), Kenyon et al. (2011) and Blair et al. (2011) as part of the same research program. To date, no comparison between single and twin muscle weight and AA concentration in muscle has been previously described for these animals.

### Intracellular free AA (FAA) profiles in fetal ST tissue

Amino acids were determined by ion-exchange chromatography using post-column derivatization with ninhydrin. Approximately 150 mg of ST tissue from each animal was homogenised in 1.75 mL of Seraprep (Pickering Laboratories, Alphatech Systems Ltd, Auckland, New Zealand) containing 20 μL of L-2-Amino-3-guanidinopropionic acid hydrochloride (25 μM/mL) as an internal standard (Calbiochem-Behring Corp., La Jolla, CA, USA). Samples were left in ice for 20 minutes, and then 40 μL 5.88 M lithium hydroxide buffer (BDH Chemical, Poole, England) added, followed by centrifugation at 8000 g for 10 minutes. After centrifugation, samples were filtered using a 0.45 μm cellulose acetate filter membrane (Advantec, Toyo Roshi, Tokyo, Japan). Supernatant was analysed for FAA using a Shimadzu LC10Ai high-performance liquid chromatography (HPLC) (Shimadzu Oceania Ltd., Auckland, New Zealand), fitted with a high-efficiency lithium-ion exchange column (3 mm ID × 150 mm; Pickering Laboratories, Shimadzu Oceania Ltd., Auckland, New Zealand) and a Pickering PCX 3100 post-column reaction module (Pickering Laboratories, Shimadzu Oceania Ltd, Auckland, New Zealand). Injected volumes were 10 μL, at a flow rate of 0.3 mL/min and a run time of 162 minutes between injections, using Li buffers as eluants and ninhydrin post-column derivatization (Csapó et al. 2008). Detection was performed at 570 nm for all FAA, except proline which was read at 440 nm. Amino acids in samples were quantified on the basis of known amounts of standards (Shimadzu Oceania, Pickering Laboratories, USA) and their retention times, using LC Solution ver. 1.22 SP1 software (Shimadzu, Kyoto, Japan).

### Statistical analysis

Fetal ST muscle weight and FAA concentration were analysed using the MIXED procedure (SAS 2006) with a linear model, which included the fixed effects of pregnancy rank (single vs. twin), ewe size (H vs. L) and ewe nutrition (A vs. M) and their two- and three-way interactions. Individual ewe tag was used as a random effect to adjust for twinning. Differences among least squares means were analysed using the PDIFF option of the MIXED procedure. To examine whether fetal ST weight was proportional to fetal weight, fetal weight was used as covariate in a separate analyses. Both adjusted and unadjusted values are presented for comparison. Sex of fetus had no effect on any traits of interest and was removed from the ST muscle and FAA models. Means are presented as least square means with least significant differences (LSD, 5%). Isoleucine, asparagine and cystine were detected in only some animals, therefore these FAA were omitted from analysis. The results for the effect of maternal size, maternal nutrition and maternal nutrition by size interaction were not included and will be presented elsewhere.

To determine the correlation between ST muscle weight and intracellular FAA concentration, partial correlations (SAS 2006) were estimated on the residual of the AA concentration and muscle mass, after accounting for the effects of pregnancy rank, maternal size and nutritional treatments and results consider all animals. For all analysis, statistical significance was set at a probability value of *P* ≤ 0.05.

## Abbreviations

AA: Amino acids
EAA: Essential amino acid
FAA: Free amino acid
NEAA: Non essential amino acid
ST: Semitendinosus
BCAA: Branched-chain amino acids.
